# Quantitative analysis of the optic nerve head parameters in patients with age-related macular degeneration

**DOI:** 10.3906/sag-1904-210

**Published:** 2019-10-24

**Authors:** Mert ŞİMŞEK, Mehmet ÇITIRIK, Serdar ÖZATEŞ, Tülay ŞİMŞEK

**Affiliations:** 1 Department of Ophthalmology, University of Health Sciences, Ulucanlar Eye Education and Research Hospital, Ankara Turkey; 2 Department of Ophthalmology, Kars Harakani State Hospital, Kars Turkey; 3 Department of Ophthalmology, Faculty of Medicine, Osmangazi University, Eskişehir Turkey

**Keywords:** Age-related macular degeneration, confocal scanning laser ophthalmoscopy, cup shape, Heidelberg retinal tomography, optic nerve head parameters

## Abstract

**Background/aim:**

To evaluate the topographic parameters of the optic disc of patients with age-related macular degeneration (AMD) by performing confocal scanning laser ophthalmoscopy.

**Materials and methods:**

This prospective study included 41 eyes of 41 patients with neovascular AMD, 56 eyes of 56 patients with nonneovascular AMD, and 48 eyes of 48 healthy control subjects. Images of the optic nerve head of all of the participants were obtained using Heidelberg retinal tomography III software 3.1. The following stereometric parameters were measured for each participant: disc area, cup area, rim area, cup volume, rim volume, cup-to-disc ratio, mean cup depth, maximum cup depth, cup shape, and mean retinal nerve fiber layer thickness.

**Results:**

The cup shape values ​​of the patients with neovascular and nonneovascular AMD were significantly different from those of the control subjects (P = 0.002 and P < 0.001, respectively). The cup-to-disc ratio was significantly higher in the patients with nonneovascular AMD when compared with the control subjects (P = 0.013), but no difference was found between the patients with neovascular AMD and the control subjects (P > 0.05). No significant differences were observed among the 3 groups with respect to the other optic disc parameters (P > 0.05).

**Conclusion:**

These data showed that the deterioration of the cup shape was an important finding in patients with AMD. Because AMD manifests with progressive ocular damage, including the optic nerve head, examination of the cup shape may be important during the follow-up of these patients.

## 1. Introduction

Age-related macular degeneration (AMD) is a leading cause of irreversible vision loss [1]. In addition to sociodemographic risk factors such as age, sex, and race, the consumption of a high-fat diet, alcohol, and antioxidant-poor diet, as well as smoking, play important roles in the etiopathogenesis of AMD. Various systemic diseases, including hypertension, hyperlipidemia, and hyperglycemia, and ocular risk factors also increase the incidence of AMD [2]. Moreover, hypermetropia, ocular melanin deficiency, and previous cataract surgery are the proven risk factors of AMD.

Morphological changes in the optic nerve head are associated with various ocular diseases, such as myopia, primary open-angle glaucoma, retinal vascular occlusion, and optic nerve diseases [3–5]. Confocal scanning laser ophthalmoscopy (also referred to as Heidelberg retinal tomography [HRT]) allows the topographic analysis of the optic nerve head and peripapillary region [6]. HRT is an important ocular examination and plays a vital role in the diagnosis and monitoring of glaucoma. Moreover, it allows noninvasive and rapid imaging of diseases involving the peripapillary area and optic nerve.

Various studies have shown that patients with AMD show disturbances in different optic disc parameters. Law et al. observed a decrease in the neuroretinal rim and an increase in the cup-to-disc ratio of patients with advanced AMD [7]. In contrast, Hall et al. observed no significant association between the presence and severity of AMD and the cup-to-disc ratio [8]. Moreover, the Eye Disease Case-Control (EDCC) Study Group showed that a high cup-to-disc ratio was associated with a reduced risk of neovascular AMD [9].

Therefore, the present study evaluated the topographic parameters of the optic disc of patients with AMD by performing HRT and compared these parameters with those of healthy control subjects. In addition, subgroup analysis was performed to compare the topographic parameters of the optic disc between patients with neovascular AMD and those with nonneovascular AMD.

## 2. Materials and methods

This prospective study included newly-diagnosed and treatment-naive AMD patients who were admitted to our retina department. Sex- and age-matched control participants were selected in the outpatient clinic. Patients with a history of ocular surgery, ocular trauma, ocular hypertension, or glaucoma, or those with first-degree relatives with primary open-angle glaucoma were excluded from the study. Patients who provided an unreliable optic disc image and those with cataract, high spherical (more than or equal ±3 diopters [D]) or cylindrical (>1 D) refractive errors, and systemic hypertension were also excluded from the study. Inclusion criteria for the control group were refraction of <3 D for myopia, <3 D for hyperopia, and <1 D for astigmatism; intraocular pressure (IOP) of <22 mmHg; no evidence of glaucomatous changes in the optic disc; no history of ocular surgery; no history of systemic diseases; and no primary open-angle glaucoma in the first-degree relatives. All the procedures performed in the study involving human participants were in accordance with the ethical standards of the institutional and/or national research committee, and with the 1964 Helsinki declaration and its later amendments or comparable ethical standards. Approval was obtained from the local ethics committee of the Numune Education and Research Hospital and written informed consent was obtained from all individual participants included in the study (Reference of review board: E-15-499). All of the patients were Turkish Caucasians.

First, the demographic data of the participants, including age and sex, were obtained. Next, all of the participants underwent a comprehensive ophthalmologic examination, including measurement of best corrected visual acuity (BCVA), using the Early Treatment Diabetic Retinopathy Study chart, and measurement of the IOP, by performing applanation tonometry, and biomicroscopic examination of the anterior and posterior segments after dilation.

Neovascular AMD was diagnosed based on the presence of choroidal neovascularization and associated manifestations, such as subretinal fluid and retinal pigment epithelial detachment [10]. Nonneovascular AMD was diagnosed based on the presence of geographic atrophy in the macula, but not extending to the optic disc area, and the absence of the clinical evidence of choroidal neovascularization [10]. The right eyes of the participants in the control group were included in the study. The clinically worse eyes of the participants in the both of the patient groups were included in the study. The optical coherence tomography and fundus autofluorescence techniques were used to confirm the diagnosis of neovascular AMD and nonneovascular AMD.

After undergoing a detailed ophthalmologic examination, all of the participants underwent confocal scanning laser ophthalmoscopy (HRT III, software version 3.1; Heidelberg Engineering GmbH, Heidelberg, Germany), which was performed by the same experienced physician who was blinded to the clinical findings of the patients. During the HRT, 3 topographic images were obtained for each eye of the patients, without pupil dilation and with a 15 × 15 field of view, under the same intensity of dim room lighting. Next, a composite image was constructed from these 3 images, which was then used for the analysis. A contour line of the disc margin was drawn manually from the inner edge of the scleral ring by placing 8–10 points. The software calculated various parameters relative to a reference plane that was 50-mm posterior to the retinal surface at the papillomacular bundle. The disc area, cup area, cup-to-disc ratio, rim area, cup volume, rim volume, mean cup depth, maximum cup depth, cup shape measurement, and mean retinal nerve fiber layer (RNFL) thickness were evaluated.

2.1. Statistical analysis 

The results of an a priori power analysis using power and sample size (PASS; version 11, NCSS, LLC, Utah, USA) calculation software required the enrolment of at least 28 eyes from each group in the study. Accordingly, the power of the study was found to be 89.7%.

Statistical analyses were performed using SPSS version 18.0 for Windows (IBM Corp., Armonk, NY, USA). The variables were evaluated using visual (histograms and probability plots) and analytical methods (the Kolmogorov–Smirnov or Shapiro–Wilk test) to determine whether or not they were normally distributed. Differences in the disc parameters among the groups were evaluated using one-way analysis of variance (ANOVA), where applicable, followed by the Bonferroni correction. Pairwise post hoc tests were performed using Tukey’s test when an overall significance was observed. P < 0.05 was considered statistically significant.

## 3. Results

This prospective study examined 41 eyes of 41 patients with neovascular AMD, 56 eyes of 56 patients with nonneovascular AMD, and 48 eyes of 48 healthy control subjects. The neovascular AMD group included 15 men (36.5%) and 26 women (63.4%), the nonneovascular AMD group included 23 men (41.0%) and 33 women (58.9%), and the control group included 18 men (37.5%) and 30 women (62.5%). The mean (± standard deviation) ages of the participants in the neovascular AMD, nonneovascular AMD, and control groups were 68.70 ± 5.22, 69.03 ± 5.25, and 67.33 ± 4.43 years, respectively. No significant differences were observed among the study groups with respect to age and sex (P = 0.202 and P = 0.931, respectively). However, the BCVA values were significantly different among the 3 groups (P < 0.001; Table 1).

**Table 1:** Demographic data of the groups.

	Neovascular AMD(n = 41)	Nonneovascular AMD(n = 56)	Control group(n = 48)	P-value
Age (years)	68.70 ± 5.22	69.03 ± 5.25	67.33 ± 4.43	0.202
Sex (m/f)	15/26	23/33	18/30	0.931
BCVA (LogMAR), (min-max)	1.08 ± 0.61(0.15–2.10)	0.74 ± 0.72(0–3.10)	0.02 ± 0.02(0–0.05)	<0.001*<0.001^a*^, <0.001^b*^, 0.004^c*^

AMD: age-related macular degeneration, BCVA: best-corrected visual acuity, m: male, f: female.P-value: One-way ANOVA for age and BCVA, chi-squared test for sex (comparison among the 3 groups).^a^Significance between the neovascular AMD and control groups (pairwise comparison).^b^Significance between the nonneovascular AMD and control groups (pairwise comparison).^c^Significance between the neovascular AMD and nonneovascular AMD groups (pairwise comparison).*Statistically significant values.

The optic disc parameters of the patients in the neovascular AMD, nonneovascular AMD, and control groups are shown in Table 2. Significant differences were observed among the groups with respect to the cup shape value and cup-to-disc ratio (one-way ANOVA, P = 0.001 and P = 0.041, respectively). In contrast, no significant differences were observed among the groups with respect to the other optic disc parameters (P > 0.05).

**Table 2:** Comparison of the optic disc parameters between the neovascular AMD, nonneovascular AMD, and control groups

	Neovascular AMD(n = 41)	Nonneovascular AMD(n = 56)	Control group(n = 48)	P-value
Disc area	2.22 ± 0.56	2.21 ± 0.39	2.23 ± 0.43	0.802
Cup area	0.42 ± 0.28	0.46 ± 0.30	0.34 ± 0.30	0.068
Rim area	1.79 ± 0.59	1.76 ± 0.36	1.89 ± 0.41	0.149
Cup volume	0.08 ± 0.08	0.08 ± 0.08	0.06 ± 0.08	0.133
Rim volume	0.55 ± 0.26	0.47 ± 0.17	0.50 ± 0.16	0.243
Cup-to-disc ratio	0.18 ± 0.12	0.21 ± 0.11	0.14 ± 0.12	0.041*0.068^a^, 0.013^b*^, 0.419^c^
Mean cup depth	0.18 ± 0.08	0.18 ± 0.07	0.15 ± 0.07	0.056
Maximum cup depth	0.54 ± 0.23	0.52 ± 0.17	0.47 ± 0.20	0.245
Cup shape	–0.18 ± 0.06	–0.18 ± 0.05	–0.23 ± 0.08	0.001*0.002^a*^, <0.001^b*^, 0.657^c^
Mean RNFL thickness	0.24 ± 0.10	0.21 ± 0.07	0.23 ± 0.05	0.062

RNFL: retinal nerve fiber layer.P-value: One-way ANOVA test (comparison among the 3 groups).^a^Significance between the neovascular AMD and control groups (pairwise comparison).^b^Significance between the nonneovascular AMD and control groups (pairwise comparison).^c^Significance between the neovascular AMD and nonneovascular AMD groups (pairwise comparison).*Statistically significant values.

Results of the pairwise analysis showed that the cup shape values of the patients with neovascular and nonneovascular AMD were significantly higher than those of the control subjects (Student’s t-test, P = 0.002 and P < 0.001, respectively). The cup-to-disc ratio of the patients with nonneovascular AMD was higher that of the control subjects (Student’s t-test, P = 0.013), but was not different between the patients with neovascular AMD and the control subjects (P > 0.05). However, a significant moderately positive correlation was observed between the cup shape value and cup-to-disc ratio of the patients with AMD (P < 0.001, r = 0.443). 

Furthermore, no significant differences were observed between the patients with neovascular and nonneovascular AMD with respect to all of the optic disc parameters (P > 0.05).

## 4. Discussion

In the present study, a significant difference was observed among the groups with respect to the cup-to-disc ratio and cup shape value. The results of the pairwise analysis showed that the cup shape values of the patients with neovascular and nonneovascular AMD were significantly higher than those of the control subjects. Moreover, the cup-to-disc ratio of the patients with neovascular and nonneovascular AMD was higher than that of the control subjects; however, this difference was significant only between the patients with nonneovascular AMD and the control subjects.

AMD is the most important cause of legal blindness in people aged ≥65 years, and its incidence increases with an increase in age [1]. Aging induces atrophy due to oxidative stress, impairs retinal pigment epithelium-Bruch’s membrane complex, and promotes extracellular deposit formation. Moreover, AMD leads to progressive vision loss because of subretinal and/or intraretinal fluid accumulation, pigment epithelial detachment, and choroidal neovascularization. Studies have reported peripapillary atrophy in the retinal pigment epithelium [11], thinning of peripapillary choroidal thickness [12], and a reduction in the ganglion cell complex thickness in patients with AMD [13,14]. Patients with advanced AMD also show optic disc changes [7].

Measurement of the cup-to-disc ratio is critical for monitoring various eye diseases, particularly those characterized by a high IOP. An increased IOP is accompanied by optic disc rim thinning, peripapillary atrophy, and an increased cup-to-disc ratio. However, both the glaucomatous process and ischemic and inflammatory processes can lead to these changes. Hayreh et al. reported high cup-to-disc ratios for patients with central retinal vein occlusion [15]. In contrast, Beck at al. showed that a decreased cup-to-disc ratio was associated with an increased risk of anterior ischemic optic neuropathy [16].

Different studies have reported different results for the association between optic disc cupping and AMD. Law et al. examined stereoscopic disc photographs and reported that patients with advanced AMD showed a decrease in the neuroretinal rim and an increase in the cup-to-disc ratio [7]. However, Hall et al. reported no significant association between the presence and severity of AMD, and the cup-to-disc ratio [8]. Similarly, Budde et al. performed a stereoscopic fundus analysis and observed no relationship between AMD and optic disc appearance [17]. The study by the EDCC Study Group, performed in 1992 and involving a large patient series, reported that the risk of neovascular AMD decreased with an increase in the cup-to-disc ratio; however, this study did not clearly define the method used for measuring the cup-to-disc ratio [9].

Recently, confocal scanning laser ophthalmoscopy has become popular because it allows quantitative analysis of the optic nerve head. This scanning method is frequently used for diagnosing and monitoring glaucoma, optic nerve pathologies, and various retinal diseases [18–20]. In the present study, the cup-to-disc ratio was higher in the patients with neovascular and nonneovascular AMD than in the control subjects, which was in contrast to that reported by the EDDC study group. However, the difference was only significant between the patients with nonneovascular AMD and the control subjects (P = 0.013). The inflammatory processes involved in AMD etiopathogenesis, which triggers cell death and progressive atrophy of the intraocular structures, may increase the cup-to-disc ratio in patients with AMD. Moreover, reduced choriocapillaris perfusion in patients with advanced AMD may play a substantial role in increasing the cup-to-disc ratio [21]. One of the strengths of the present study was the inclusion of patients with neovascular and nonneovascular AMD and the comparison of the cup-to-disc ratio between the patients with AMD, and between these patients and the control subjects.

The maximum cup depth, rim volume, and cup shape, which are measured by performing HRT, are the global parameters of optic disc injury [22]. Uchida et al. suggested that the cup shape and cup-to-disc ratio have the highest diagnostic value in patients with advanced glaucoma [23]. Consistently, the cup shape value and cup-to-disc ratio were significantly different among the groups in the present study (P = 0.001 and P = 0.041, respectively). The results of the subgroup analysis showed that the cup shape values of the patients with AMD were significantly higher than those of the control subjects. Moreover, a significantly moderately positive correlation was observed between the cup-to-disc ratio and cup shape value in patients with AMD (P < 0.001, r = 0.443).

Durukan et al. evaluated the topographic parameters of the optic nerve head in healthy subjects and reported that the cup shape value was the only parameter that was not influenced by age, sex, disc area, laterality, and refractive error [24]. Therefore, they suggested that the cup shape was potentially the most important HRT measurement for comparing different cases, because it was independent of the other factors. These data, together with the findings of the present study, suggested that the difference in the cup shape values between the patients with AMD and the control subjects is an important parameter for monitoring disease progression in these patients.

Saarela et al. stated that progressive RNFL loss was only correlated with the cup shape among the topographic parameters of the optic disc [25]. In the present study, progression of RNFL loss was not evaluated because the baseline parameters of the patients were considered. Although no significant correlation was observed between the cup shape value and mean RNFL value in the present study, the mean RNFL value was not significantly different between the patients with AMD and the control subjects.

The present study was one of the few studies to evaluate the parameters of the optic nerve head in patients with AMD. However, additional studies involving a greater number of patients should be performed to more accurately determine the differences in these parameters.

In conclusion, the present study showed a significant change in the topographic parameters of the optic nerve head, particularly in the cup-to-disc ratio and cup shape value, in patients with AMD when compared with those in the control subjects, and suggested that the cup shape is a valuable parameter for monitoring patients with AMD during follow-up.
